# Explaining the intention of dental health personnel to report suspected child maltreatment using a reasoned action approach

**DOI:** 10.1186/s12913-019-4330-8

**Published:** 2019-07-22

**Authors:** Ingfrid Vaksdal Brattabø, Ragnhild Bjørknes, Kyrre Breivik, Anne Nordrehaug Åstrøm

**Affiliations:** 1 Oral Health Centre of Expertise in Western Norway, Hordaland, Pb. 2354 Møllendal, 5867 Bergen, Norway; 2 Department of Health Promotion and Development, Faculty of Psychology, University of Bergen, Pb. 7807, 5020 Bergen, Norway; 3 Department of Clinical Dentistry, Faculty of Medicine, University of Bergen, Norway, Pb. 7804, 5020 Bergen, Norway; 4 Regional Centre for Child and Youth Mental Health and Child Welfare, NORCE Norwegian Research Centre, Bergen, Norway, Pb 7810, 5020 Bergen, Norway

**Keywords:** Child maltreatment, Child abuse, Child welfare services, Dental auxiliaries, Mandatory reporting, Oral health, Reasoned action approach, Structural equation modelling

## Abstract

**Background:**

This study provides an empirical test of the reasoned action approach (RAA) socio-cognitive theory with the aim of 1) predicting the intention of public dental health personnel (PDHP) to report suspected child-maltreatment to child welfare services (CWS); 2) estimating the effects of the theoretical constructs of RAA, including experiential and instrumental attitudes, injunctive and descriptive norms, and perceived capacity and autonomy regarding PDHP’s behavioural intentions; and 3) exploring whether the RAA operates equivalently (i.e., is invariant) in male and female providers.

**Methods:**

This national cross-sectional study was conducted in Norway. An electronic survey was distributed to 1542 dentists and dental hygienists working in the public dental health service. The survey included RAA items constructed in accordance with the recommendations for the RAA model. Structural equation modelling (SEM) was used to identify factors derived from the theory of RAA to predict PDHP reporting intentions.

**Results:**

A total of 77.8% (1200) of those surveyed responded to the survey. The present study provided support for the utility of the RAA across both male and female providers in predicting their intention to report suspected child-maltreatment to the CWS. The final modified SEM model revealed that instrumental attitudes and perceived behavioural control (based on merged capacity and autonomy parameters) were the strongest predictors of intention to report, followed by the reporting of descriptive norms, injunctive norms and experiential attitudes. These factors explained 63.6% of the observed variance in the reporting intention.

**Conclusions:**

The large amount of explained variance suggests that RAA is a well-functioning theory that predicts PDHP’s reporting intentions to CWS across gender, and gives an understanding of the socio-cognitive factors involved. To strengthen reporting intention among dental personnel, this study suggests educators should focus on the value and positive consequences of reporting, the resources available and how to overcome obstacles; attention to normative expectations and individuals’ feelings about reporting may also be helpful.

**Electronic supplementary material:**

The online version of this article (10.1186/s12913-019-4330-8) contains supplementary material, which is available to authorized users.

## Background

Victims of child maltreatment have an augmented risk for major psychiatric and medical disorders [1–5]. The scope and severity of these disorders are likely to increase with the duration and severity of maltreatment. For this reason, the early detection of victimized children is an important objective worldwide [6–9]. In Norway, public dental health personnel (PDHP) are mandated through the Norwegian Health Personnel Act, § 33, to report to the child welfare service (CWS) when there is reason to believe that a child is or will be abused, subjected to serious deficiencies in daily care or other serious neglect.

This obligation goes above and beyond health personnel’s duty of confidentiality §21. [10]. Failure to fulfil the Norwegian Health Personnel Act, § 33, can result in administrative reactions from the Norwegian Board of Health Supervision. As in the other Nordic countries, Norwegian children are offered free dental service at public dental health service (PDHS) locations throughout their childhood and adolescence (0–18 years) [11]. With a dental attendance rate close to 100%, the Norwegian PDHP meets most children and adolescents on a regular basis, making the PDHS an important arena for the detection of child maltreatment. Statistics Norway reports that the CWS in Norway received 58 580 reports of concern in 2017, of which 768 came from the PDHS [12]. According to a Norwegian national study, 60% of PDHP reported to have sent at least one report of concern to the CWS during their career [13]. In regard to PDHP’s reporting frequency throughout career, adjusted analysis revealed no significant differences in incidence rate ratio between dentists and dental hygienists or across age groups, while females were less likely to report to CWS than males. Further, PDHP working in municipalities with 10.000 or less inhabitants were less likely to report than their colleagues working in larger municipalities. [13]. While most reports of concern to CWS from PDHS relate to oral conditions, failure to attend and not being brought to dental appointments, reports of concern are also sent due to suspicion of neglect, physical, psychological and sexual abuse [14]. Dental personnel suspect and identify a variety of child maltreatments [14]. The awareness and knowledge regarding detection of child maltreatment and the role of dental personnel has increased in recent years. As a consequence a new paragraph §1-3c was added to the Norwegian Dental Health Service Act in 2018, stating that the PDHS should be able to prevent, detect and avert violence and sexual abuse [11].

Yet, underreporting of suspected child maltreatment is a challenge among dental health personnel world-wide [13, 15–20]. The national study among PDHP in Norway revealed that 32% of the dental health personnel investigated failed to report suspected child maltreatment to the CWS one or several times during their career [13]. Such findings are consistent with those from other countries and imply that steps should be taken to strengthen the reporting accuracy of suspected child maltreatment [15, 16, 19–22]. Underreporting of child maltreatment can have major consequences for the child, its family and the society at large. The gap between suspicion of child maltreatment and reporting to CWS needs to be closed. Hence, there is a need to understand which factors that inhibit and promote dental personnel’s reporting. The effective promotion of mandatory reporting obligations in the PDHS may require a thorough understanding of the socio-cognitive factors underlying the decision of dental health personnel to report suspected maltreatment to the CWS. Previous studies have identified reporting barriers among dental health personnel, such as uncertainty regarding their observations and signs of child maltreatment, lack of knowledge regarding reporting procedures, and fear of consequences to child and dental personnel [15, 19–21]. However, in spite of their importance, socio-cognitive factors have not been sufficiently investigated. While conceptual frameworks have been used to examine reporting of child maltreatment among teachers and nurses [23–25], to our knowledge, no theory driven studies have been conducted for dental health personnel. A socio-cognitive model that adequately explains variance in intended reporting of suspected child maltreatment to the CWS could be an important tool in order to develop an effective behaviour change program for dental health personnel. Although such a socio-cognitive model has yet to be validated among dental health personnel.

A socio cognitive model of the attitude – behaviour relationship, the theory of planned behaviour (TPB) [26] has been applied across various study populations, and behavioural domains to predict intention and subsequent behaviour [27–30]. According to the TPB, behavioural intention is the immediate predictor of actual behaviour [31, 32]. Intention, in turn, is predicted by attitudes, subjective norms and perceived behavioural control (PBC). Attitudes reflect a favourable or unfavourable evaluation of a particular behaviour. Subjective norms refer to perceived social pressures to perform a given behaviour, and perceived behavioural control reflect the perceived ease or difficulty associated with performing a particular behaviour. Finally, attitudes, subjective norms and perceived behavioural control are underpinned by behavioural, normative and control beliefs, respectively [26]. The TPB hypothesizes that attitudes, subjective norms and perceived behavioural control influence the behaviour indirectly through behavioural intentions and that perceived behavioural control influences behaviour directly whenever the behaviour is not under complete volitional control [33]. There is considerable empirical support for the TPB across various health related behaviours, including health screening behaviours [26, 27]. Two meta-analyses have revealed that, overall, the TPB explains between 39 and 44.3% and 19.3–27% of the variance in intention and subsequent behaviour, respectively [27, 34].

**Fig. 1:**
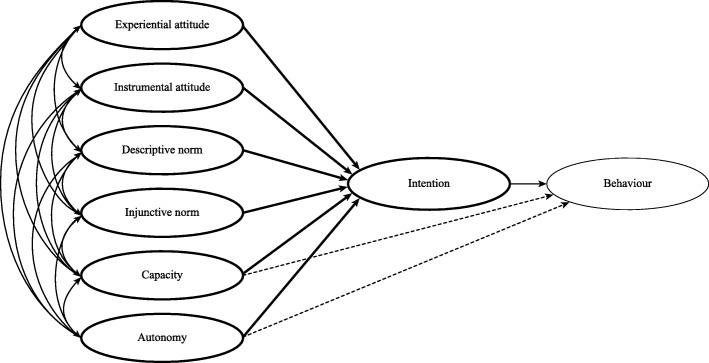
Original RAA model. The bold parts show the hypothesized model measured in this study

Many studies have argued that attitudes, subjective norms and perceived behavioural control reflect separate binary subcomponents, as shown in Fig. 1 [35–37]. This conceptual development of the TPB is variously referred to as an augmented TPB model, a two-factor model, and the reasoned action approach (RAA) [35, 38–41]. The RAA suggests that: Attitude consist of the subcomponents experiential (i.e., affective component of attitude) and instrumental (i.e., cognitive component of attitude) attitudes. Perceived norm consists of the subcomponents injunctive (i.e., the perceived social approval of others) and descriptive norms (i.e., perceptions of what others do). Perceived behavioural control consists of the subcomponents capacity to perform the behaviour (based on the ease or difficulty of the behaviour) and autonomy (their perception of their control over the behaviour). According to the RAA, intention is the immediate predictor of behaviour, whereas each attitudinal, normative and control sub-component predicts intention directly. In addition, capacity and autonomy predicts behaviour directly when the behaviour is not under volitional control [41, 42]. Moreover, the RAA predicts that the relative importance of the theoretical constructs on behavioural intention may vary across various behaviours and groups of participants. See Fig. 1 for details of the original RAA model. Thus, the RAA provides a unique opportunity to identify the relative importance of each specific subcomponent (i.e. experiential and instrumental attitude, injunctive and descriptive norm, capacity and autonomy) as predictors of intention and behaviour. The RAA has received empirical support across risk and protective health related behaviours. A meta-analysis covering risk behaviours like smoking and taking illegal drugs, protective behaviours like physical activity and dieting, and a range of different health related behaviours like health screening and blood donating revealed that the RAA explained 58.7 and 32.3% of the variance in intention and behaviour, respectively [41]. Moreover, experiential and instrumental attitudes and capacities were found to be the strongest predictors of intention, while injunctive and descriptive norms were more modest predictors [41]. Few studies have focused on health and dental health personnel’s professional behaviour using a socio-cognitive approach [43–47]. It seems worthwhile to investigate whether the predictive utility of the RAA can be generalized to dental health personnel’s intention to report child maltreatment in the primary dental health care setting. Further, Brattabø et al. [13], found no significant differences between males and females, in regard to dental personnel’s incidence rate ratio for reporting to CWS in recent years (2012–2014), while the incidence rate ratio for reporting throughout career was significant lower for females compared to men [13]. Due to this it is also important to assess whether the RAA operates equivalently (i.e., is invariant) across males and females.

This study provides an empirical test of the RAA with the aim of 1) predicting the intention of PDHP to report suspected child maltreatment to CWS; 2) estimating the effects of the theoretical constructs of RAA, including experiential and instrumental attitudes, injunctive and descriptive norms, and perceived capacity and autonomy regarding PDHP’s reporting intentions; and 3) exploring whether the RAA operates equivalently (i.e., is invariant) in male and female providers.

Consistent with the conceptualization of the RAA [38], we hypothesized that a model with the following characteristics would fit the variables measuring experiential (i.e., affective component of attitude) and instrumental (i.e., cognitive component of attitude) attitudes, injunctive (i.e., the perceived social approval of others) and descriptive (i.e., perceptions of what others do) norms, capacity (i.e. the ease or difficulty to perform the behaviour) and autonomy (i.e., their perception of their control over the behaviour) and intention (i.e., to report suspected child maltreatment); the model would include seven factors corresponding to the measuring items used in scoring each theoretical construct.

## Methods

A census of all the registered public dentists and dental hygienists employed in the PDHS in 18 of the 19 counties in Norway were asked to take part in a national cross-sectional study. The last county was not included, as it was used in the pilot study. The names and contact information of the dental professionals were collected from the chiefs of the Norwegian PDHS’s, who also allowed the survey to be administered during business hours. The study’s objectives and a link to an electronic questionnaire, containing an informed consent page, were sent by electronic mail to all the registered public dentists and dental hygienists a total of 1542 dentists and dental hygienists. The estimated completion time of the survey was 30–40 min (The questionnaire is available as Additional file 1). The survey was approved and registered by the Ombudsman of the Norwegian Social Science Data Services (NSD) (Reference number: 40581/4/LH/LR) who administered the questionnaire distribution and data collection, in November 2014. Non-responders were given reminders after two, four and seven weeks.

The questionnaire was developed in three stages to ensure that the instrument was well suited to the Norwegian public dental health context. First, the semantics and content of the questions were assessed, and the questions were translated and back translated from Norwegian to English. Second, PDHP with experience in survey research and clinical work reviewed the questionnaire. Third, a pilot study in one county (*n* = 176) was conducted.

The questionnaire incorporated each theoretical construct of the RAA model in terms of the experiential and instrumental attitudes, injunctive and descriptive norms, and the capacity and autonomy and intention assessed in relation to the likelihood of reporting suspected child abuse or neglect in the following 12-month period. The questions related to each theoretical construct of the RAA were constructed in accordance with the principle of compatibility and based on recommendations for the reasoned action approach model proposed by Ajzen and Fishbein in 2010 [38]. In line with the recommendations that each predictor should be self-referent and measured at the same specificity as the target behaviour, the elements of the target (reporting suspected child maltreatment), the action (sending a report of concern to CWS), the context (the public dental health service) and the time (during the next 12 months) were considered [38]. Experiential attitude (i.e., tapping affective aspects of behavioural beliefs) and instrumental attitude (i.e., tapping cognitive aspects of behavioural beliefs), capacity (i.e., based on the ease or difficulty to report suspected child maltreatment) and autonomy (i.e., their perception of their control in regard to report suspected child maltreatment) and intention to report suspected child maltreatment were each measured by four items. Injunctive norm (i.e., the perceived social approval of others in regard to report suspected child maltreatment) and descriptive norm (i.e., perceptions of what others do when they suspect child maltreatment), were measured by five items each, giving a total of 30 items, see Table 2. Responses were provided on five point Likert scales (with possible responses ranging from 1 to 5), with varying response options (i.e., quite unlikely/quite likely, very difficult/very easy, totally disagree/ totally agree).Respondents’ previous experience with suspecting and reporting child maltreatment was assessed along with their demographical characteristics, including gender, age, occupation, years of working experience in the PDHS, number of patients treated last 12 months, county and size of municipality where dental clinic was located. Additional information regarding the DPHP reporting experience can be found in Brattabø et al. 2016 [13].

### Statistics

The Statistical Package for Social Sciences version 22 (SPSS Inc., Chicago, IL, USA) was used for descriptive statistics in terms of frequency % (n) and mean (SD) distributions. Mplus version 7.4 (Muthen & Muthen 1998–2015) was used to test the structural equation models (SEM).

The original hypothesized RAA model (See Fig. 1, in bold) was tested using a two-step modelling approach (Kline, 2011). In the first step, the hypothesized RAA model was re-specified as a correlated factor model to test the adequacy of the measurement model. In the second step, a full structural regression model was conducted to test the plausibility of the postulated RAA model (including potential modifications based on the findings detailed in step 1). Modification indices were used to test for sources of misfit. Multiple group analyses were used in both steps to test for invariance across gender. A prerequisite for exploring whether the predictive paths are gender invariant (step 2) is that the measurement model (step 1) is both configural (equal form) and metric invariant (equal factor loadings) across men and women [48]. Configural invariance was examined by testing the fit of the measurement model separately for women and men. When testing for metric invariance, the fit of the models for which the loadings on each specific factor were held equal between genders was compared to a baseline 2-group configural model in which the same parameters (except for the identification items) were free to vary. The model was assumed to be non-invariant if the change in the chi- square was significant (as tested by changes in Satorra-Bentler scaled χ2 [49]) and the decrease in CFI was larger than 0.002 [50] compared to the baseline model.

The maximum likelihood estimator with robust standard errors (MLR) was used to take into account the non-normally distributed data. To measure how well the model fit the sample data, the overall goodness of fit was assessed by the Chi-square test (x^2^), the standardized root mean squared residual (SRMR), the root mean square error of approximation (RMSEA) and the comparative fit index (CFI). A good fit between the measurement model and the data is indicated by a Chi square test with a statistically insignificant result at the *p* < 0.05 threshold. However, as the Chi-square test is highly dependent on the sample size, it is possible to detect trivial problems in large samples. We therefore put more emphasis on the alternative fit indices when judging the model fit. An acceptable and good fit is indicated by an SRMR < 0.08 and < 0.05, an RMSEA < 0.08 and < 0.06 and a CFI > 0.90 and > 0.95 [51, 52].

Regarding missing data, 17 of the 1200 cases had missing on all included variables and were therefore excluded. The sample size varied between 1183 and 1113 (see Tables 1 and 2). The MLR estimators, including full information maximum likelihood (FIMLs), were used to handle the remaining missing data [53]. This is the default method for handling missing when using the maximum likelihood estimator in Mplus 7.4 and is generally superior to standard ad hoc missing data routines such as the mean replacement, pairwise deletion and listwise deletion [54].

**Table 1:** Frequency distribution % (n) characteristics of the studied public dental health personnel

Characteristics	Categories	Dentists	Dental hygienists	Total
Gender		% (n)	% (n)	% (n)
	Female	72.1 (554)	98.6 (341)	80.3 (895)
	Male	27.9 (214)	1.4 (5)	19.7 (219)
Age				
	20–39 years	57.3 (440)	41.6 (144)	52.4 (584)
	40+ years	42.7 (328)	58.4 (202)	47.6 (530)
Working experience at PDHS				
	1–10 years	66.0 (507)	45.4 (157)	59.6 (664)
	11+ years	34.0 (261)	54.6 (189)	40.4 (450)
Number of patients < 19 years.*				
	0–500	47.4 (364)	24.1 (83)	40.2 (447)
	501 − +	52.6 (404)	75.9 (262)	59.8 (666)

* last 12 months

**Table 2:** Descriptive statistics for RAA measurement model

Latent factor		Item	N	Question	Answers	Cronb. alpha	Mean		Skewness		Kurtosis	
					Valued 1–5 (very difficult = 1 – very easy = 5)		Statistic	Std. dev	Statistic	Std.error	Statistic	Std.error
Experiential attitude		1	1183	To send a report of concern on suspicion of child abuse or neglect the following 12 months is	very difficult, difficult, neither/nor, easy, very easy	.814	2.64	.884	.538	.071	.180	.142
		2	1181	To send a report of concern on suspicion of child abuse or neglect the following 12 months is	very onerous, onerous, neither/nor, simple, very simple		2.72	.770	.504	.071	.324	.142
		3	1183	To send a report of concern on suspicion of child abuse or neglect the following 12 months is	very unpleasant, unpleasant, neither/nor, pleasant, very pleasant		2.33	.649	.309	.071	.657	.142
		4	1183	To send a report of concern on suspicion of child abuse or neglect the following 12 months is	very demanding, demanding, neither/nor, no problem, absolutely no problem		2.52	.749	.556	.071	.506	.142
Instrumental attitude		5	1183	To send a report of concern on suspicion of child abuse or neglect the following 12 months is	totally unimportant, unimportant, neither/nor, important, very important	.825	4.72	.495	−1.851	.071	5.586	.142
		6	1183	To send a report of concern on suspicion of child abuse or neglect the following 12 months is	completely useless, useless, neither/nor, useful, very useful		4.44	.585	−.547	.071	−.346	.142
		7	1183	To send a report of concern on suspicion of child abuse or neglect the following 12 months is	totally wrong, wrong, neither/nor, right, completely right		4.66	.506	−1.050	.071	.147	.142
		8	1181	To send a report of concern on suspicion of child abuse or neglect the following 12 months is	very unwise, unwise, neither/nor, wise, very wise		4.40	.634	−.670	.071	.072	.142
Descriptive norm				The following persons do always send a report of concern on suspicion of child abuse or neglect								
		9	1166	my colleagues at the dental clinic	totally disagree, disagree, neither/nor, agree, totally agree	.903	3.66	.894	−.411	.072	.038	.143
		10	1165	my boss at the dental clinic	totally disagree, disagree, neither/nor, agree, totally agree		3.71	.906	−.432	.072	.130	.143
		11	1165	most persons in my situation	totally disagree, disagree, neither/nor, agree, totally agree		3.62	.799	−.219	.072	.031	.143
		12	1165	most people who are important to me	totally disagree, disagree, neither/nor, agree, totally agree		3.57	.801	−.061	.072	.040	.143
		13	1165	most persons like me	totally disagree, disagree, neither/nor, agree, totally agree		3.67	.789	−.143	.072	.028	.143
Injunctive norm				If I during the coming 12 months suspects child abuse or neglect								
		14	1168	do my colleagues at the dental clinic think that I should send a report of concern	totally disagree, disagree, neither/nor, agree, totally agree	.921	4,32	.765	−1.438	.072	3.327	.143
		15	1167	does my boss at the dental clinic think that I should send a report of concern	totally disagree, disagree, neither/nor, agree, totally agree		4.38	.742	−1.418	.072	3.126	.143
		16	1167	does the public dental leader group at the county level think that I should send a report of concern	totally disagree, disagree, neither/nor, agree, totally agree		4.36	.784	−1.489	.072	3.163	.143
		17	1167	most persons important to me think that I should send a report of concern	totally disagree, disagree, neither/nor, agree, totally agree		4.30	.773	−1.265	.072	2.456	.143
		18	1168	is it expected of me that I should send a report of concern	totally disagree, disagree, neither/nor, agree, totally agree		4.45	.673	−1.326	.072	2.985	.143
Capacity	PBC			If I, during the coming 12 months, suspects child abuse or neglect								
		19^b^	1167	I am very unsure if I am able to send a report of concern	totally disagree, disagree, neither/nor, agree, totally agree	.744	4.01	.946	−1.097	.072	1.092	.143
		20	1168	I am absolutely confident I can send a report of concern	totally disagree, disagree, neither/nor, agree, totally agree.		4.00	.885	−1.036	.072	1.296	.143
		21	1167	I have full opportunity to send a report of concern	totally disagree, disagree, neither/nor, agree, totally agree.		4.38	.699	−1.589	.072	4.904	.143
		22^b^	1167	it would be difficult to send a report of concern	totally disagree, disagree, neither/nor, agree, totally agree		3.60	1.057	−.458	.072	−.571	.143
Autonomy				If I, during the coming12 months, suspects child abuse or neglect								
		23	1167	there are few outside events that can prevent me from sending a report of concern	totally disagree, disagree, neither/nor, agree, totally agree		3.66	1.026	−.693	.072	.071	.143
		24	1168	I have complete control over sending a report of concern	totally disagree, disagree, neither/nor, agree, totally agree		3.63	.942	−.451	.072	−.282	.143
		25^b^	1167	sending a report of concern is beyond my control	totally disagree, disagree, neither/nor, agree, totally agree		4.21	.749	−1.034	.072	1.849	.143
		26^a^	1166	It is entirely up to me whether I will send a report of concern or not	totally disagree, disagree, neither/nor, agree, totally agree.		3.64	1.154	−.705	.072	−.373	.143
Intention		27	1155	In the next 12 months I intend to send a report of concern to the CWS if I suspect child abuse or neglect	totally disagree, disagree, neither/nor, agree, totally agree	.759	4.38	.859	−2.099	.072	5.592	.144
		28	1155	If I in the coming 12 months suspect child abuse or neglect, I will send a report of concern	completely unsure, unsure, neither/nor, sure, completely sure		4.23	.790	−1.086	.072	1.636	.144
		29	1155	If I during the next 12 months gets suspicious of child abuse or neglect I want to send a report of concern	totally disagree, disagree, neither/nor, agree, totally agree		4.47	.685	−1.568	.072	4.150	.144
		30	1149	if you during the next 12 months is concerned for a child (regarding child abuse or neglect) how unlikely or likely is it that you will send a report of concern?	quite unlikely, unlikely, neither/nor, likely, quite likely		4.12	.622	−.304	.072	.534	.144

^a^Item 26 was deleted

^b^Items 19, 22 and 25 were negatively loaded, and their values were reversed

## Results

A total of 1200 of the eligible 1542 (RR 77.8%) dentists and dental hygienists responded to our survey. In accordance with gender and professional distribution in the Norwegian PDHS, 19.7 and 80.3% of the respondents were men and women, respectively, while 31.1% were dental hygienists and 68.9% were dentists. Among the respondents, 82.9% had examined more than 250 children and adolescents < 19 years of age during the previous 12 months. The mean working experience of the respondents was 11.9 (SD = 11.2) years (Table 1).

Throughout their career, 32.6% of the respondents had failed to report suspected child abuse or neglect, with a mean of 2.3 (SD =1.8) failures. In contrast, 60% of the respondents were experienced reporters, having sent at least one reports of concern, with a mean of 3.6 (SD = 3.4)) reports [13].

Table 2 shows the descriptive statistics for each of the 30 items measuring the RAA constructs in terms of mean, standard deviation and skewness. The range for the items was between 1 and 5, with low values indicating negative or weak cognitions and high values indicating positive or strong cognitions. As shown in Table 2, while items 1–4 (experiential attitude) had mean values < 2.8, item 5–30 (instrumental attitudes, descriptive and injunctive norm and capacity and autonomy) all had mean values > 3.5.

### Step 1. Measurement model

The initially proposed correlated seven-factor model (Model 1) (experiential and instrumental attitude, injunctive and descriptive norm, capacity and autonomy and intention) lacked an adequate fit to the data on most fit indices employed, see Table 3. The analysis further revealed that the capacity and autonomy factors were very highly correlated (standardized correlation coefficient = 0.839, SE = 0.051, *P* < 0.001), at above 0.800 and close to the cut off measure of 0.850, indicating poor discriminant validity between the two latent variables [55]. In addition, preliminary analysis showed that multicollinearity would lead to inflated standard errors in the paths in the full structural equation model [56]. The capacity and autonomy factors were therefore merged into one latent factor, labelled perceived behavioural control, including 8 indicators (item 19–26), reducing the number of latent factors in the measurement model from seven to six. Merging these two factors was supported by explorative factor/component analyses. Horn’s Parallel analysis suggested six factors and an explorative factor analysis (geomin (oblique) rotation) gave generally good support for the modified six-factor solution. Increasing the number of factors to seven did furthermore not lead to separate capacity and autonomy factors as hypothesized by the original model. The re-estimation of the correlated six-factor model (Model 2) also lacked an adequate fit to the data however, see Table 3.

**Table 3:** Overall goodness of fit indices for the RAA measurement model (model 1–4) and full structural model (model 5)

Fit indices	Model 1	Model 2	Model 3	Model 4	Model 5
X^2^	1875.570	1902.915	1136.095	1071.041	1071.041
DF	384, *P* < 0.001	390, *P* < 0.001	385, *P* < 0.001	357, *P* < 0.001	357, *P* < 0.001
RMSEA	0.057	0.057	0.041	0.041	0.041
90% CI for RMSEA	0.055–0.060	0.055–0.060	0.038–0.043	0.038–0.044	0.038–0.044
CFI	0.884	0.882	0.941	0.943	0.943
SRMR	0.053	0.054	0.046	0.046	0.046

Modification indices suggested some misfit in the model and that the model fit could be improved by allowing for correlated residuals between the items in the descriptive norm factor (item 9 with item 10, item 12 with item 13), the perceived behavioural control factor (item 20 with item 21) and the intention factor (item 27 with item 29). In addition, item 22 was cross-loaded on the experiential attitude factor in addition to the perceived behavioural control factor. Re-estimation of the modified six factor model (Model 3) provided an adequate fit, see Table 3. The standardized factor loadings for the correlated six factor model (Model 3) revealed that all items, except one, loaded significantly *P* < 0.001 on their respective latent variables, all with factor loadings > 0.300. The exception was the autonomy item 26) (“If I, during the coming twelve months, suspects child abuse or neglect, it is entirely up to me whether I will send a report of concern or not.”). This item was loaded 0.100 on the merged perceived behavioural control factor. Due to the low factor loading, the autonomy item 26 was dropped, and the model was re-estimated.

The final modified measurement model (Model 4) achieved an adequate fit, see Table 3. As shown in Table 4, all items loaded significantly (*p* < .001) and in the expected direction on their respective latent variables. The statistically significant standardized loadings ranged from 0.332 to 0.894. All inter-factor correlations were below the cut-off point of 0.850 for the standardized correlation coefficient. The standardized correlation coefficient ranged from 0.120, SE 0.035 *P* = 0.001 for injunctive norms and experiential attitudes to 0.679, SE 0.037 *P* < 0.001 for intention and perceived behavioural control, indicating discriminant validity between the latent variables [55].

**Table 4:** Standardized factor loadings for the RAA measurement model 4

Latent factor		Item	Stand. factor loadings	Std. error
Experiential attitude				
		1	0.817	0.017
		2	0.802	0.017
		3	0.657	0.025
		4	0.627	0.025
		22	0.369	0.033
Instrumental attitude				
		5	0.695	0.036
		6	0.718	0.020
		7	0.802	0.019
		8	0.753	0.020
Descriptive norm				
		9	0.762	0.019
		10	0.732	0.021
		11	0.866	0.015
		12	0.810	0.020
		13	0.763	0.023
Injunctive norm				
		14	0.856	0.018
		15	0.894	0.014
		16	0.825	0.020
		17	0.841	0.018
		18	0.767	0.026
Capacity	PBC			
		19^b^	0.637	0.037
		20	0.696	0.036
		21	0.466	0.049
		22^b^	0.413	0.038
Autonomy				
		23	0.332	0.038
		24	0.629	0.024
		25^b^	0.459	0.035
		^a^		
Intention				
		27	0.516	0.041
		28	0.785	0.027
		29	0.683	0.034
		30	0.636	0.030

^a^Item 26 was deleted

^b^Items 19, 22 and 25 were negatively loaded, and their values were reversed

All loadings were significant at *P* < 0.001

Configural invariance across the genders was supported, as Model 4 had an adequate fit for both females (X^2^ = 909.080, d.f. = 357, *P* < 0.001, RMSEA 0.042, 90% CI for RMSEA 0.038–0.045, CFI = 0.944, SRMR = 0.049) and males (X^2^ = 597.199, d.f. = 357, *P* < 0.001, RMSEA 0.055, 90% CI for RMSEA 0.048–0.063, CFI = 0.908, SRMR = 0.061). Metric invariance (equal factor loading) was also obtained for each factor (results not shown), thus demonstrating that the size of the predictive paths could be compared between men and women in step 2.

### Step 2. The full structural equation model

**Fig. 2:**
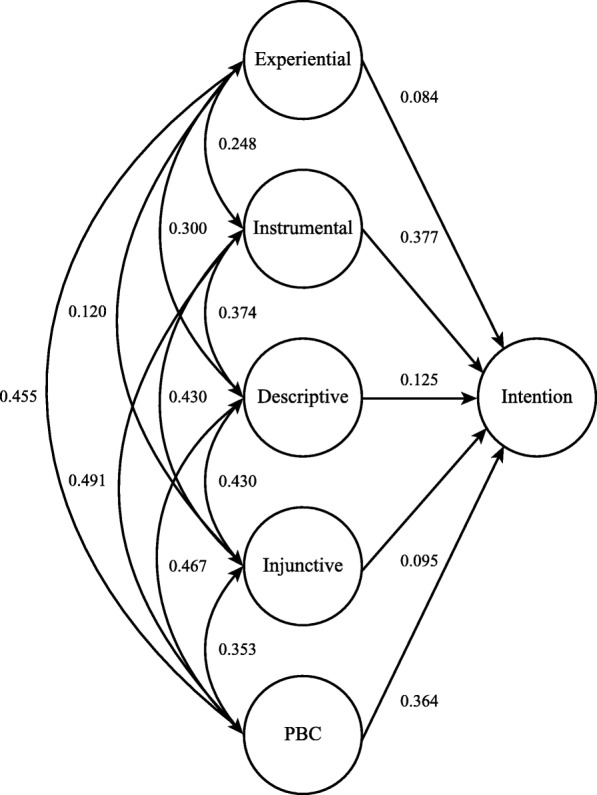
SEM model 5 (Standardized coefficients)

Based on the adequate fit of the six-factor model (Model 4), a full structural equation model was conducted to estimate the fit of the structural model and the relationships among the latent constructs, see Fig. 2. The full structural equation model (Model 5) achieved a good model fit, see Table 3. The analysis revealed that having an instrumental attitude (standardized beta = 0.377, SE 0.047, *P* < 0.001) and perceived behavioural control (Standardized beta = 0.364, SE 0.049, P < 0.001) were the strongest predictors of intention, followed by descriptive norms (standardized beta = 0.125, SE 0.043, *P* < 0.01), injunctive norms (standardized beta = 0.095, SE 0.040, *P* < 0.05) and experiential attitudes (standardized beta = 0.084, SE 0.036, *P* < 0.05). The full structural equation model (Model 5) revealed that the modified RAA model (capacity and autonomy merged) could explain 63.6% of the variance in the behavioural intention (*R*^2^ = 0.636, SE 0.050 *P* < 0.001).

Multi-group analyses of the full structural equation model did support the invariant regression paths across gender, as the fit of the model did not significantly worsen when each of the predictive paths were constrained to be equal compared to when they were free to vary across the genders, see Table 5.

**Table 5:** Test for invariance of the predictive paths across gender

Equality constraint	Δ Satorra-Bentler Scaled Chi Square	Δdf	Probability	ΔCFI
Instrumental attitude ➔ Intention	0.73+	1	0.39	< 0.01
Experiential attitude ➔ Intention	1.79	1	0.18	< 0.01
Injunctive norm ➔ Intention	0.16	1	0.69	< 0.01
Descriptive norm ➔ Intention	0.36	1	0.55	< 0.01
Perceived behavioural control ➔ Intention	0.34	1	0.56	< 0.01

+ Worsening of the fit when the path was constrained to be equal compared to when it was free to vary across gender

## Discussion

To our knowledge, the present study is the first to explain the intentions of dental health personnel regarding reporting suspected child maltreatment using a socio-cognitive theoretical framework (RAA).

The findings of the present study provided support for the utility of the RAA across males and females in predicting dental health personnel’s intention to report suspicion of child maltreatment to the CWS. The modified RAA model, demonstrated a good fit to the data, and explained 63.6% of the variance in the behavioural intentions. This suggests that the RAA is a well-functioning theory in order to predict and explain dental health personnel’s professional reporting behaviour. In accordance with the RAA, we found that the instrumental attitude (i.e., cognitive aspects of behavioural beliefs), perceived behavioural control (i.e., perception of control and capacity to report suspicion of child maltreatment), descriptive norm (i.e., perceptions of what others do), injunctive norm (i.e., the perceived social approval of others) and experiential attitude (i.e., affective aspects of behavioural beliefs) were, in descending order of importance, significant predictors of intended reporting behaviour. The present findings were consistent with a previous review using the RAA to predict risk- and protective health-related behaviours, implying that the RAA also can be used to predict reporting behaviour [41]. Although the RAA and the original TPB differ in terms of the number of predictors of behavioural intention, the explained variance in behavioural intention obtained in this study (63.6%) compared well with the those reported in studies using the TPB to predict health care personnel’s professional behaviour [44, 57, 58]. In meta-analyses, the RAA and the TPB accounted for 59 and 44% of the variance in the behavioural intentions, respectively [34, 41]. Altogether, the findings and explained variance indicate that the RAA functions well in order to assess and predict reporting intention among dental health personnel.

In the present study, the instrumental attitude (i.e., cognitive aspects of behavioural beliefs) emerged as the strongest determinant of intended reporting behaviour. This implies that the decision to report was strongly based on the anticipated benefits of performing that behaviour, for the child and society. This is consistent with previous TPB-based studies focusing dental personnel’s professional behaviour, which found that attitudes are a strong predictor of intensions related to fissure sealing and oral radiographs [44, 45, 47]. A recent TPB study predicting dentists’ intended delivery of a variety of prevention activities in regard to diet, alcohol and smoking, revealed that attitudes were an important predictor of their intentions to perform preventive behaviours [46]. Experiential attitudes (i.e., affective aspects of behavioural beliefs) turned out to be the weakest predictor of reporting intention. This suggests that even though reporting could be demanding or challenging, it has only a minor influence on dental health personnel’s reporting intention. This finding was at odds with previous studies using RAA, for which experiential attitudes have been found to be one of the main predictors of health-related intention and behaviour [41, 59–61]. Nevertheless, the relative effect of the theoretical constructs is expected to vary according to the type of behaviour and the participants under study [62]. The professional behaviour investigated in this study might be categorized as a detection behaviour which is suggested to differ from risk- and protective health behaviours [59]. Consistent with this reasoning, Conner et al. (2015) provided empirical support for the predictive effect of affective or experiential attitudes on risk- and protective health-related behaviours, whereas no such effects on the category of detection behaviour were seen.

In accordance with a meta analytical review of studies using the RAA [41], PDHP’s perception of control and capacity (PBC) turned out to be a strong predictor of the intended reporting behaviour. This suggests that not only beliefs about the positive consequences of reporting behaviour but also beliefs about difficulties and facilitating aspects associated with reporting should be targeted in educational messages that aim to enhance the intended reporting behaviour. The strong effect of perceived behavioural control is consistent with the results of previous studies that have identified actual barriers towards reporting suspected child maltreatment among professionals required to report suspected abuse, emphasizing a lack of knowledge about the signs of abuse and referral procedures in addition to the negative consequences for the patient, as important barriers [15, 19, 63].

Both descriptive (i.e., perceptions of what others do) and injunctive (i.e., the perceived social approval of others) norms turned out to be independent, albeit rather weak predictors of dental health personnel’s’ intended reporting behaviour. This suggests that dentists and dental hygienists are guided not only by normative expectations from others but also by what significant others actually do regarding reporting behaviour. As mandated through the Norwegian Health Personnel Act, normative beliefs may have connotations to dental health personnel’s moral obligations, responsibilities or personal standards in relation to reporting child maltreatment. Meta analytical reviews have also shown that descriptive norms add to the prediction of health related behaviours independent of the attitudes, subjective norms and perceived behavioural controls [64]. Consistent with the results of the present study, Godin et al. (1999) found normative beliefs to be a predictor of dentists’ intention to provide dental care to HIV+/AIDS infected patients [58]. Furthermore, in studies using RAA and TPB, descriptive and injunctive norms are often observed as weak or non- significant predictors of intention. Importantly, injunctive norms have traditionally turned out to be weaker predictors of behavioural intention than attitudes and perceived behavioural controls [27].

The present findings should be interpreted in the context of the strengths and limitations of this study. Being cross-sectional and relying on self-reports, conclusions about cause-and-effect relationships are difficult to draw, and there is a risk of reporting bias as respondents might be those who are interested in the topic. It is also important to be aware that some behaviors might be driven differently between cultures. Thus, one should be careful to extrapolate the findings to other cultures and populations. Another limitation is related to intentions being the final dependent variable and not actual reporting behaviour, as hypothesized by the RAA. Future studies should therefore have a longitudinal design and investigate both intended and actual reporting behaviour. However, the present study was national and included a census of public dentists and dental hygienists in Norway. Moreover, the high 78% response rate [13] reduces the possibility that missing responses have seriously biased the collected data on the intended reporting behaviour [65, 66], although social desirability might have biased the answers. In addition, the present study utilizes a powerful multivariable statistical technique testing the RAA model overall rather than the coefficients individually [67]. In contrast to traditional multivariate methods, SEM is well fit to address complex behaviours, as it allows for the simultaneous analysis of both the observed and latent variables, their relationships and the model fit. Furthermore, SEM also accounts for measurement errors by providing estimates of error variance parameters while simultaneously estimating the modelled path coefficients [68]. The application of SEM improves the conceptual understanding of the RAA as a structural and measurement model.

Although information about the performance of the RAA across age groups and other socio-demographic characteristics of the study population would have been of interest, the present multi-group analysis by gender strengthened our findings to some extent. The present findings have implications for dentistry and educational institutions, providing guidance for the development of future interventions.

The study suggests relatively strongly that educational messages intending to strengthen dental health personnel’s intention to report suspected maltreatment would benefit from an emphasis on the benefits of such reports for the child, its family and the society at large. There should also be an emphasis on the specifics about how to make such a report and that dental health personnel are capable and permitted to do this. Moreover, the reporting intention might be further strengthen by educational messages focusing on the normative aspects regarding reporting of child maltreatment, in terms of clarifying that reporting is socially accepted, expected and the right thing to do. In addition one should acknowledge that reporting often is hard and demanding but useful.

## Conclusions

This study provided support for the utility of a modified RAA model across gender in predicting dental health personnel’s intention to report suspected child maltreatment to the CWS. Norwegian PDHP’s intention to report suspected child maltreatment was mostly based on considerations of likely positive cognitive consequences of performance, required resources and potential obstacles, as well as normative expectations and affective attitude, in that order. To strengthen reporting intention among dental personnel, this study suggests educators should focus on the value and positive consequences of reporting, the resources available and how to overcome obstacles; attention to normative expectations and individuals’ feelings about reporting may also be helpful.

Emphasizing these factors in the future training and education of dental health personnel might strengthen the reporting intention of suspected child maltreatment and contribute to reduce the gap between suspicion and reporting. Future studies should incorporate a measure of observed behaviour. A detailed analysis of the belief structure underlying attitudes, norms and perceived behavioural control may extend the applicability of the RAA model.

## Additional file

Additional file 1: Tannhelse og barnevern – samhandling til beste for barnet. Questionnaire regarding dental personnel’s suspicion of child maltreatment and reporting to child welfare services. The questionnaire was sent to dental hygienists and dentists in the public dental health service in Norway 2014. The questionnaire is previously published in Brattabø et al. 2018 [14]. (PDF 254 kb)

## Data Availability

The dataset will not be made available, as more articles are to be published based on this dataset.

## Abbreviations

CFI: Comparative Fit Index
CWS: Child Welfare Service
GEE: Generalized Estimating Equations
MLR: Maximum Likelihood Estimator with Robust Standard Errors
NSD: Norwegian Social Science Data Services
PBC: Perceived Behavioural Control
PDHP: Public Dental Health Personnel
PDHS: Public Dental Health Service
RAA: Reasoned Action Approach
RMSEA: Root Mean Square Error of Approximation
SEM: Structural equation modelling
SRMR: Standardized Root Mean Squared Residual
TPB: Theory of Planned Behaviour
