# Supplementary material for: Explaining the intention of dental health personnel to report suspected child maltreatment using a reasoned action approach
Source: BMC Health Serv Res. 2019 Jul 22;19:507. doi: 10.1186/s12913-019-4330-8 (PMC6647276; doi:10.1186/s12913-019-4330-8)
Supplement: Supplementary file 1 — Tannhelse og barnevern – samhandling til beste for barnet. Questionnaire regarding dental personnel’s suspicion of child maltreatment and reporting to child welfare services. The questionnaire was sent to dental hygienists and dentists in the public dental health service in Norway 2014. The questionnaire is previously published in Brattabø et al. 2018 [14]. (PDF 254 kb) [file 12913_2019_4330_MOESM1_ESM.pdf]

Velkommen til spørreundersøkelsen:
Tannhelse og barnevern -samhandling til beste for barnet

Hjertelig takk for at du vil delta i studien.

Dine erfaringer som tannhelsepersonell er et viktig bidrag for at vi sammen kan generere forskningsbasert kunnskap som kommer tannhelsetjenesten og utsatte barn til gode.

Spørreundersøkelsen er begrenset til å ta ca. 30 til 40 minutter. Noen spørsmål kan oppleves like, men de fanger opp ulike aspekter og alle svar er viktige.

Vi setter stor pris på at du deltar i denne studien, sammen kan vi øke kunnskapen!

Hjertelig takk!

UNIVERSITETET I BERGEN
HEMIL-senteret
Senter for forskning om helsefremmende arbeid, miljø og livsstil

Tannhelsetjenestens
kompetansesenter
Vest / Hordaland

De første spørsmålene er knyttet opp mot dine holdninger til å sende bekymringsmelding til barnevernet ved mistanke om barnemishandling eller omsorgssvikt.

	helt uenig	uenig	verken enig eller uenig	enig	helt enig
Som tannhelsepersonell føler jeg et profesjonelt ansvar for å sende bekymringsmelding ved mistanke om barnemishandling eller omsorgssvikt	◯	◯	◯	◯	◯
Personlig føler jeg et etisk ansvar for å sende bekymringsmelding ved mistanke om barnemishandling eller omsorgssvikt	◯	◯	◯	◯	◯
Jeg føler et yrkesansvar for å sende en bekymringsmelding ved mistanke om barnemishandling eller omsorgssvikt	◯	◯	◯	◯	◯

Dersom jeg i løpet av de neste 12 måneder, sendte bekymringsmelding ved mistanke om barnemishandling eller omsorgssvikt, ville jeg …

	helt uenig	uenig	verken enig eller uenig	enig	helt enig
følt at bekymringsmeldingen ville beskytte barnet mot videre skade	◯	◯	◯	◯	◯
vært bekymret for den umiddelbare sikkerheten til barnet	◯	◯	◯	◯	◯
vært bekymret for den negative påvirkningen dette ville få for mitt forhold til barnet	◯	◯	◯	◯	◯
vært veldig stresset for å ha sendt en bekymringsmelding	◯	◯	◯	◯	◯
vært bekymret for hvor mye av min arbeidstid dette ville ta	◯	◯	◯	◯	◯
følt at jeg hadde handlet riktig overfor barnet	◯	◯	◯	◯	◯
vært i tvil om barnevernstjenesten er i stand til å respondere passende på min bekymringsmelding	◯	◯	◯	◯	◯
vært bekymret for at min bekymringsmelding ikke er godt nok begrunnet i henhold til bevis	◯	◯	◯	◯	◯
følt at jeg bidrog til å redusere omfanget av barnemishandling og omsorgssvikt i vårt lokalsamfunn	◯	◯	◯	◯	◯
følt at dette ville gjøre situasjonen verre for barnet	◯	◯	◯	◯	◯
følt at jeg gjorde min plikt	◯	◯	◯	◯	◯
følt at jeg har tatt barnet på alvor	◯	◯	◯	◯	◯

Dersom jeg i løpet av de neste 12 måneder, sendte en bekymringsmelding ved mistanke om barnemishandling eller omsorgssvikt, og bekymringen viste seg å være grunnløs…

	helt uenig	uenig	verken enig eller uenig	enig	helt enig
ville jeg bekymret meg for de negative konsekvensene dette kunne få for meg	◯	◯	◯	◯	◯
ville jeg bekymret meg for de negative konsekvensene dette kunne få for tannklinikken	◯	◯	◯	◯	◯
ville jeg bekymret meg for de negative konsekvensene dette kunne få for barnets familie	◯	◯	◯	◯	◯

Dersom jeg i løpet av de neste 12 måneder, sendte en bekymringsmelding ved mistanke om at noen i barnets familie utøvde barnemishandling eller omsorgssvikt, ville jeg…

	helt uenig	uenig	verken enig eller uenig	enig	helt enig
vært bekymret for hvilken innvirkning dette ville få for mitt forhold til barnets foreldre	◯	◯	◯	◯	◯
tenkt at jeg handlet riktig	◯	◯	◯	◯	◯
vært bekymret for hvilke konsekvenser dette ville få for barnets foreldre og andre familiemedlemmer	◯	◯	◯	◯	◯
tenkt at dette, på lang sikt, ville bidra til å forbedre situasjonen for barnets familie	◯	◯	◯	◯	◯

Dersom jeg i løpet av de neste 12 måneder unnlot å sende en bekymringsmelding ved mistanke om barnemishandling eller omsorgssvikt..

	helt uenig	uenig	verken enig eller uenig	enig	helt enig
ville jeg blitt så bekymret for barnet at jeg ville hatt problemer med å få sove om natten	◯	◯	◯	◯	◯
ville jeg bekymret meg for hvilke juridiske konsekvenser dette kunne få for meg	◯	◯	◯	◯	◯
og barnet fortsatte å bli mishandlet eller utsatt for omsorgssvikt, ville jeg ha angret på at jeg ikke hadde meldt inn min mistanke	◯	◯	◯	◯	◯

De påfølgende spørsmål omhandler hva du tenker rundt det å sende en bekymringsmelding ved mistanke om at en av dine pasienter er utsatt for barnemishandling eller omsorgssvikt de neste 12 måneder.

Å sende bekymringsmelding ved mistanke om barnemishandling eller omsorgssvikt de neste 12 mnd. er...

- ◯ helt uviktig
- ◯ uviktig
- ◯ verken uviktig eller viktig
- ◯ viktig
- ◯ veldig viktig

Å sende bekymringsmelding ved mistanke om barnemishandling eller omsorgssvikt de neste 12 mnd er...

- helt unyttig
- unyttig
- verken nyttig eller unyttig
- nyttig
- veldig nyttig

Å sende bekymringsmelding ved mistanke om barnemishandling eller omsorgssvikt de neste 12 mnd. er...

- helt feil
- feil
- verken feil eller rett
- riktig
- helt riktig

Å sende bekymringsmelding ved mistanke om barnemishandling eller omsorgssvikt den neste 12 mnd. er...

- veldig uklokt
- uklokt
- verken uklokt eller klokt
- klokt
- veldig klokt

Å sende bekymringsmelding ved mistanke om barnemishandling eller omsorgssvikt de neste 12 mnd. er...

- veldig vanskelig
- vanskelig
- verken lett eller vanskelig
- lett
- veldig lett

Å sende bekymringsmelding ved mistanke om barnemishandling eller omsorgssvikt de neste 12 mnd. er...

- veldig belastende
- belastende
- verken belastende eller enkelt
- enkelt
- veldig enkelt

Å sende bekymringsmelding ved mistanke om barnemishandling eller omsorgssvikt de neste 12 mnd. er...

- veldig ubehagelig
- ubehagelig
- verken ubehagelig eller behagelig
- behagelig
- veldig behagelig

Å sende bekymringsmelding ved mistanke om barnemishandling eller omsorgssvikt de neste 12 mnd. er...

- veldig krevende
- krevende
- verken krevende eller en smal sak
- en smal sak
- en veldig smal sak

De neste spørsmål omhandler din oppfatning av hva hva personer i ditt sosiale miljø og arbeidsmiljø mener når det gjelder å sende bekymringsmelding.

Dersom jeg i løpet av de neste 12 måneder mistenker barnemishandling eller omsorgssvikt...

	helt uenig	uenig	verken enig eller uenig	enig	helt enig
mener mine kollegaer på tannklinikken at jeg skal sende bekymringsmelding	○	○	○	○	○
synes min klinikksjef at jeg skal sende bekymringsmelding	○	○	○	○	○
mener ledergruppen i tannhelsetjenesten (fylkestannlege/direktør/overtannleger etc.) at jeg skal sende bekymringsmelding	○	○	○	○	○
mener de fleste personer som er viktige for meg at jeg skal sende bekymringsmelding	○	○	○	○	○
er det forventet av meg å sende bekymringsmelding	○	○	○	○	○

Dersom jeg i løpet av de neste 12 måneder sendte en bekymringsmelding ved mistanke om barnemishandling eller omsorgssvikt, ville jeg fått støtte fra...

	helt uenig	uenig	verken enig eller uenig	enig	helt enig
mine kollegaer på tannklinikken	○	○	○	○	○
min klinikksjef (evt annen nærmeste leder)	○	○	○	○	○
ledergruppen i tannhelsetjenesten (fylkestannlege/direktør/overtannlege etc.)	○	○	○	○	○
de fleste personer som er viktige for meg	○	○	○	○	○
personer som er lik meg	○	○	○	○	○

De påfølgende spørsmål omhandler hvordan du opplever det å sende en bekymringsmelding ved mistanke om at en av dine pasienter er utsatt for barnemishandling eller omsorgssvikt de neste 12 måneder.

Dersom jeg i løpet av de neste 12 måneder får mistanke om barnemishandling eller omsorgssvikt...

	helt uenig	uenig	verken uenig eller enig	enig	helt enig
er jeg svært usikker på om jeg er i stand til å sende en bekymringsmelding	○	○	○	○	○
er jeg helt trygg på at jeg kan sende en bekymringsmelding	○	○	○	○	○
har jeg full mulighet til å sende en bekymringsmelding	○	○	○	○	○
vil det være veldig vanskelig å sende en bekymringsmelding	○	○	○	○	○

Dersom jeg i løpet av de neste 12 måneder, får mistanke om barnemishandling eller omsorgssvikt...

	helt uenig	uenig	verken enig eller uenig	enig	helt enig
er det få utenforliggende faktoere som kan forhindre meg i å sende en bekymringsmelding	○	○	○	○	○
har jeg full kontroll på det å sende en bekymringsmelding	○	○	○	○	○
ligger avgjørelsen om å sende en bekymringsmelding utenfor min kontroll	○	○	○	○	○
er det helt opp til meg om jeg vil sende en bekymringsmelding eller ikke	○	○	○	○	○

De påfølgende spørsmål omhandler din oppfatning av i hvilken grad viktige personer sender en bekymringsmelding ved mistanke om at en av deres pasienter er utsatt for barnemishandling eller omsorgssvikt de neste 12 måneder.

Følgene personer sender alltid bekymringsmelding ved mistanke om barnemishandling eller omsorgssvikt.

	helt uenig	uenig	verken enig eller uenig	enig	helt enig
mine kollegaer på tannklinikken	○	○	○	○	○
min klinikksjef	○	○	○	○	○
de fleste personer i min situasjon	○	○	○	○	○
de fleste personer som er viktige for meg	○	○	○	○	○
de fleste personer lik meg	○	○	○	○	○

Tannhelsepersonell opplever ofte at det kan være vanskelig å sende en bekymringsmelding når man mistenker barnemishandling eller omsorgssvikt. De neste spørsmål omhandler hvordan du opplever det å sende en bekymringsmelding i ulike situasjoner.

Vennligst kryss av hvor vanskelig eller enkelt det vil være for deg å sende en bekymringsmelding, i løpet av de neste 12 måneder, ved mistanke om barnemishandling eller omsorgssvikt i påfølgende situasjoner.

	veldig vanskelig	vanskelig	verken enkelt eller vanskelig	enkelt	veldig enkelt
når din mistanke er basert på opplysninger fra et barn	◯	◯	◯	◯	◯
når din mistanke er basert på mange bevis	◯	◯	◯	◯	◯
når din mistanke er basert på lite bevis	◯	◯	◯	◯	◯
når det er mange andre oppgaver som krever din tid	◯	◯	◯	◯	◯
når du føler at din klinikksjef (evt annen nærmeste overordnede) ikke støtter deg i å sende en bekymringsmelding	◯	◯	◯	◯	◯
når du føler at dine kollegaer ikke støtter deg i å sende bekymringsmelding	◯	◯	◯	◯	◯
når du kjenner barnets foreldre utenom tannklinikken	◯	◯	◯	◯	◯

Av ulike grunner er det ofte svært vanskelig å avgjøre om en skal sende bekymringsmelding eller ikke. De neste spørsmål omhandler din intensjon og spesifikke fremtidsplaner når det gjelder å sende bekymringsmelding ved mistanke om barnemishandling og omsorgssvikt.

I de neste 12 måneder har jeg til hensikt å sende bekymringsmelding til barnevernet, dersom jeg får mistanke om barnemishandling eller omsorgssvikt.

- ◯ helt uenig
- ◯ uenig
- ◯ verken enig eller uenig
- ◯ enig
- ◯ helt enig

Dersom jeg i de neste 12 måneder får mistanke om barnemishandling eller omsorgssvikt, vil jeg sende bekymringsmelding.

- ◯ helt usikker
- ◯ usikker
- ◯ verken sikker eller usikker
- ◯ sikker
- ◯ helt sikker

Dersom jeg i løpet av de neste 12 måneder får mistanke om barnemishandling eller omsorgssvikt, ønsker jeg å sende en bekymringsmelding.

- helt uenig
- uenig
- verken uenig eller enig
- enig
- helt enig

De neste spørsmål omhandler din mulighet for å kunne oppdage tegn og varselsignal på barnemishandling og omsorgssvikt.

I de neste 12 måneder, hvor usikker eller sikker føler du deg på å kunne oppfatte tegn på...

	veldig usikker	usikker	verken sikker eller usikker	sikker	veldig sikker
fysisk mishandling?	○	○	○	○	○
seksuelt misbruk?	○	○	○	○	○
psykisk mishandling?	○	○	○	○	○
vanskjøtsel?	○	○	○	○	○

I de neste 12 måneder, hvor usikker eller sikker føler du deg på å kunne oppfatte tegn på barnemishandling og omsorgssvikt dersom...

	veldig usikker	usikker	verken sikker eller usikker	sikker	veldig sikker
du har mange pasienter som trenger ekstra oppfølging?	○	○	○	○	○
du ikke har kollegaer tilgjengelig som kan assistere deg?	○	○	○	○	○
du er overarbeidet?	○	○	○	○	○
du har det veldig travelt?	○	○	○	○	○

De neste spørsmål omhandler dine fremtidige planer, ved mistanke om at en av dine pasienter er utsatt for barnemishandling eller omsorgssvikt.

Dersom du i løpet av de neste 12 måneder er bekymret for et barn, hvor usannsynlig eller sannsynlig er det at du kommer til å...

	helt usannsynlig	usannsynlig	verken sannsynlig eller usannsynlig	sannsynlig	helt sannsynlig
diskutere din bekymring med din klinikksjef (nærmeste leder)?	◯	◯	◯	◯	◯
diskutere din bekymring med dine kollegaer?	◯	◯	◯	◯	◯
diskutere din bekymring med andre aktører (helsestasjon, lege, skole, barnehage)?	◯	◯	◯	◯	◯
diskutere din bekymring med barnevernet?	◯	◯	◯	◯	◯
søke informasjon om hvordan gjenkjenne indikasjoner på barnemishandlig og omsorgssvikt gjennom faglitteratur, internett, etc.?	◯	◯	◯	◯	◯
sende bekymringsmelding?	◯	◯	◯	◯	◯

De neste spørsmål omhandler dine tidligere erfaringer med å rapportere mistanke om barnemishandling og omsorgssvikt. Her menes de tilfeller du har blitt bekymret og det er blitt sendt en bekymringsmelding på bakgrunn av din bekymring (innbefatter også dersom klinikksjef/sekretær/ressursperson evt. andre har sendt på vegne av deg).

I løpet av den tiden du har arbeidet som tannhelsepersonell, har du sendt bekymringsmelding ved mistanke om barnemishandling eller omsorgssvikt?

Her menes de tilfeller der det er blitt sendt en bekymringsmelding på bakgrunn av din bekymring(innbefatter også dersom klinikksjef/sekretær/ evt. andre har sendt på vegne av deg).

◯ ja

Hvor mange ganger har du sendt bekymringsmelding? Skriv inn siffer [ ]

◯ nei

Ble noen av disse bekymringsmedlingene sendt i tidsrommet fra og med 2012 og frem til i dag?

◯ ja

◯ nei

Hvor mange bekymringsmeldinger har du sendt i tidsrommet fra og med 2012 og frem til i dag?

Her menes de tilfeller der det er blitt sendt en bekymringsmelding på bakgrunn av din bekymring (innbefatter også dersom klinikksjef/sekretær evt. andre har sendt på vegne av deg).

- en bekymringsmelding
- to bekymringsmeldinger
- tre bekymringsmeldinger
- fire bekymringsmeldinger
- fem bekymringsmeldinger
- seks bekymringsmeldinger
- syv bekymringsmeldinger
- åtte bekymringsmeldinger
- ni bekymringsmeldinger
- ti bekymringsmeldinger eller flere

De neste spørsmålene omhandler den første bekymringsmeldingen du sendte fra og med 2012.

Hvilket kjønn hadde barnet på den første bekymringsmeldingen du sendte?

- gutt
- jente

Hvilken alder hadde barnet på den første bekymringsmeldingen du sendte?

- 0 - 3 år
- 4 - 7 år
- 8 - 11 år
- 12 - 15 år
- 16 -

Hadde den ene eller begge foreldrene til barnet du sendte første bekymringsmelding på utenlandsk opprinnelse?

- [ ] ja
- [ ] nei
- [ ] vet ikke

Hva var årsaken til den første bekymringsmeldingen du sendte?

Flere kategorier kan velges.

- ☐ mistanke om fysisk mishandling
- ☐ mistanke om seksuelt misbruk
- ☐ mistanke om psykisk mishandling
- ☐ mistanke om vanskjøtsel
- ☐ gjentagende ikke møtt til time
- ☐ grav karies
- ☐ gingivitt
- ☐ mangelfull hygiene
- ☐ sår og lesjoner i munnhulen
- ☐ traume
- ☐ andre orale funn

vennligst noter

- ☐ behandlingsvegring
- ☐ samspill med foresatte
- ☐ unormal oppførsel hos barnet
- ☐ annet

vennligst noter

Hvilken tilbakemelding har du fått fra barnevernet på den første bekymringsmeldingen du sendte?

- ○ barnevernet har åpnet undersøkelse, og iverksett tiltak
- ○ barnevernet har åpnet undersøkelse, men henlagt senere
- ○ barnevernet har åpnet undersøkelse, men ikke gitt tilbakemelding om det er iverksatt tiltak eller om saken er henlagt
- ○ barnevernet har ikke åpnet undersøkelse. Saken henlagt
- ○ barnevernet har ikke gitt noen tilbakemelding
- ○ annet

vennligst noter

- ○ vet ikke

De neste spørsmålene omhandler den andre bekymringsmeldingen du sendte fra og med 2012.

Hvilket kjønn hadde barnet på den andre bekymringsmeldingen du sendte?

- jente
- gutt

Hvilken alder hadde barnet på den andre bekymringsmeldingen du sendte?

- 0 - 3 år
- 4 - 7 år
- 8 - 11 år
- 12 - 15 år
- 16 -

Hadde den ene eller begge foreldrene til barnet du sendte <u>andre</u> bekymringsmelding på utenlandsk opprinnelse?

- ☐ ja
- ☐ nei
- ☐ vet ikke

Hva var årsaken til den andre bekymringsmeldingen du sendte?

Flere kategorier kan velges.

- ☐ mistanke om fysisk mishandling
- ☐ mistanke om seksuelt misbruk
- ☐ mistanke om psykisk mishandling
- ☐ mistanke om vanskjøtsel
- ☐ gjentagende ikke møtt til time
- ☐ grav karies
- ☐ gingivitt
- ☐ mangelfull hygiene
- ☐ sår og lesjoner i munnhulen
- ☐ traume
- ☐ andre orale funn

vennligst noter

- ☐ behandlingsvegring
- ☐ samspill med foresatte
- ☐ unormal oppførsel hos barnet
- ☐ annet

vennligst noter

Hvilken tilbakemelding har du fått fra barnevernet på den andre bekymringsmeldingen du sendte?

- ○ barnevernet har åpnet undersøkelse, og iverksett tiltak
- ○ barnevernet har åpnet undersøkelse, men henlagt senere
- ○ barnevernet har åpnet undersøkelse, men ikke gitt tilbakemelding om det er iverksatt tiltak eller om saken er henlagt
- ○ barnevernet har ikke åpnet undersøkelse. Saken henlagt
- ○ barnevernet har ikke gitt noen tilbakemelding
- ○ annet

vennligst noter

- ○ vet ikke

De neste spørsmålene omhandler den tredje bekymringsmeldingen du sendte fra og med 2012.

Hvilket kjønn hadde barnet på den tredje bekymringsmeldingen du sendte?

- jente
- gutt

Hvilken alder hadde barnet på den tredje bekymringsmeldingen du sendte?

- 0 - 3 år
- 4 - 7 år
- 8 - 11 år
- 12 - 15 år
- 16 -

Hadde den ene eller begge foreldrene til barnet du sendte tredje bekymringsmelding på utenlandsk opprinnelse?

- ☐ ja
- ☐ nei
- ☐ vet ikke

Hva var årsaken til den tredje bekymringsmeldingen du sendte?

Flere kategorier kan velges.

- ☐ mistanke om fysisk mishandling
- ☐ mistanke om seksuelt misbruk
- ☐ mistanke om psykisk mishandling
- ☐ mistanke om vanskjøtsel
- ☐ gjentagende ikke møtt til time
- ☐ grav karies
- ☐ gingivitt
- ☐ mangelfull hygiene
- ☐ sår og lesjoner i munnhulen
- ☐ traume
- ☐ andre orale funn

vennligst noter

- ☐ behandlingsvegring
- ☐ samspill med foresatte
- ☐ unormal oppførsel hos barnet
- ☐ annet

vennligst noter

Hvilken tilbakemelding har du fått fra barnevernet på den tredje bekymringsmeldingen?

- ○ barnevernet har åpnet undersøkelse, og iverksett tiltak
- ○ barnevernet har åpnet undersøkelse, men henlagt senere
- ○ barnevernet har åpnet undersøkelse, men ikke gitt tilbakemelding om det er iverksatt tiltak eller om saken er henlagt
- ○ barnevernet har ikke åpnet undersøkelse. Saken henlagt
- ○ barnevernet har ikke gitt noen tilbakemelding
- ○ annet

vennligst noter

- ○ vet ikke

De neste spørsmålene omhandler den fjerde bekymringsmeldingen du sendte fra og med 2012.

Hvilket kjønn hadde barnet på den fjerde bekymringsmeldingen du sendte?

- jente
- gutt

Hvilken alder hadde barnet på den fjerde bekymringsmeldingen du sendte?

- 0 - 3 år
- 4 - 7 år
- 8 - 11 år
- 12 - 15 år
- 16 -

Hadde den ene eller begge foreldrene til barnet du sendte fjerde bekymringsmelding på utenlandsk opprinnelse?

- ☐ ja
- ☐ nei
- ☐ vet ikke

Hva var årsaken til den fjerde bekymringsmeldingen du sendte?
Flere kategorier kan velges.

- ☐ mistanke om fysisk mishandling
- ☐ mistanke om seksuelt misbruk
- ☐ mistanke om psykisk mishandling
- ☐ mistanke om vanskjøtsel
- ☐ gjentagende ikke møtt til time
- ☐ grav karies
- ☐ gingivitt
- ☐ mangelfull hygiene
- ☐ sår og lesjoner i munnhulen
- ☐ traume
- ☐ andre orale funn

vennligst noter

- ☐ behandlingsvegring
- ☐ samspill med foresatte
- ☐ unormal oppførsel hos barnet
- ☐ annet

vennligst noter

Hvilken tilbakemelding har du fått fra barnevernet på den fjerdebekymringsmeldingen du sendte?

- ○ barnevernet har åpnet undersøkelse, og iverksett tiltak
- ○ barnevernet har åpnet undersøkelse, men henlagt senere
- ○ barnevernet har åpnet undersøkelse, men ikke gitt tilbakemelding om det er iverksatt tiltak eller om saken er henlagt
- ○ barnevernet har ikke åpnet undersøkelse. Saken henlagt
- ○ barnevernet har ikke gitt noen tilbakemelding
- ○ annet

vennligst noter

- ○ vet ikke

De neste spørsmålene omhandler den femte bekymringsmeldingen du sendte fra og med 2012.

Hvilket kjønn hadde barnet på den femte bekymringsmeldingen du sendte?

- ◯ jente
- ◯ gutt

Hvilken alder hadde barnet på den femte bekymringsmeldingen du sendte?

- ◯ 0 - 3 år
- ◯ 4 - 7 år
- ◯ 8 - 11 år
- ◯ 12 - 15 år
- ◯ 16 -

Hadde den ene eller begge foreldrene til barnet du sendte femte bekymringsmelding på utenlandsk opprinnelse?

- ☐ ja
- ☐ nei
- ☐ vet ikke

Hva var årsaken for den femte bekymringsmeldingen du sendte?

Flere kategorier kan velges.

- ☐ mistanke om fysisk mishandling
- ☐ mistanke om seksuelt misbruk
- ☐ mistanke om psykisk mishandling
- ☐ mistanke om vanskjøtsel
- ☐ gjentagende ikke møtt til time
- ☐ grav karies
- ☐ gingivitt
- ☐ mangelfull hygiene
- ☐ sår og lesjoner i munnhulen
- ☐ traume
- ☐ andre orale funn

vennligst noter

- ☐ behandlingsvegring
- ☐ samspill med foresatte
- ☐ unormal oppførsel hos barnet
- ☐ annet

vennligst noter

Hvilken tilbakemelding har du fått fra barnevernet på den femte bekymringsmeldingen du sendte?

- ○ barnevernet har åpnet undersøkelse, og iverksett tiltak
- ○ barnevernet har åpnet undersøkelse, men henlagt senere
- ○ barnevernet har åpnet undersøkelse, men ikke gitt tilbakemelding om det er iverksatt tiltak eller om saken er henlagt
- ○ barnevernet har ikke åpnet undersøkelse. Saken henlagt
- ○ barnevernet har ikke gitt noen tilbakemelding
- ○ annet

vennligst noter

- ○ vet ikke

De neste spørsmålene omhandler den sjette bekymringsmeldingen du sendte fra og med 2012.

Hvilket kjønn hadde barnet på den sjette bekymringsmeldingen du sendte?

- ○ jente
- ○ gutt

Hvilken alder hadde barnet på den sjette bekymringsmeldingen du sendte?

- ○ 0 - 3 år
- ○ 4 - 7 år
- ○ 8 - 11 år
- ○ 12 - 15 år
- ○ 16 -

Hadde den ene eller begge foreldrene til barnet du sendte sjette bekymringsmelding på utenlandsk opprinnelse?

- ☐ ja
- ☐ nei
- ☐ vet ikke

Hva var årsaken til den sjette bekymringsmeldingen du sendte?

Flere kategorier kan velges.

- ☐ mistanke om fysisk mishandling
- ☐ mistanke om seksuelt misbruk
- ☐ mistanke om psykisk mishandling
- ☐ mistanke om vanskjøtsel
- ☐ gjentagende ikke møtt til time
- ☐ grav karies
- ☐ gingivitt
- ☐ mangelfull hygiene
- ☐ sår og lesjoner i munnhulen
- ☐ traume
- ☐ andre orale funn

vennligst noter

- ☐ behandlingsvegring
- ☐ samspill med foresatte
- ☐ unormal oppførsel hos barnet
- ☐ annet

vennligst noter

Hvilken tilbakemelding har du fått fra barnevernet på den sjette bekymringsmeldingen du sendte?

- ○ barnevernet har åpnet undersøkelse, og iverksett tiltak
- ○ barnevernet har åpnet undersøkelse, men henlagt senere
- ○ barnevernet har åpnet undersøkelse, men ikke gitt tilbakemelding om det er iverksatt tiltak eller om saken er henlagt
- ○ barnevernet har ikke åpnet undersøkelse. Saken henlagt
- ○ barnevernet har ikke gitt noen tilbakemelding
- ○ annet

vennligst noter

- ○ vet ikke

De neste spørsmålene omhandler den syvende bekymringsmeldingen du sendte fra og med 2012.

Hvilket kjønn hadde barnet på den syvende bekymringsmeldingen du sendte?

- jente
- gutt

Hvilken alder hadde barnet på den syvende bekymringsmeldingen du sendte?

- 0 - 3 år
- 4 - 7 år
- 8 - 11 år
- 12 - 15 år
- 16 -

Hadde den ene eller begge foreldrene til barnet du sendte syvende bekymringsmelding på utenlandsk opprinnelse?

- ☐ ja
- ☐ nei
- ☐ vet ikke

Hva var årsaken til den syvende bekymringsmeldingen du sendte?

Flere kategorier kan velges.

- [ ] mistanke om fysisk mishandling
- [ ] mistanke om seksuelt misbruk
- [ ] mistanke om psykisk mishandling
- [ ] mistanke om vanskjøtsel
- [ ] gjentagende ikke møtt til time
- [ ] grav karies
- [ ] gingivitt
- [ ] mangelfull hygiene
- [ ] sår og lesjoner i munnhulen
- [ ] traume
- [ ] andre orale funn

vennligst noter

- [ ] behandlingsvegring
- [ ] samspill med foresatte
- [ ] unormal oppførsel hos barnet
- [ ] annet

vennligst noter

Hvilken tilbakemelding har du fått fra barnevernet på den syvende bekymringsmeldingen du sendte?

- ( ) barnevernet har åpnet undersøkelse, og iverksett tiltak
- ( ) barnevernet har åpnet undersøkelse, men henlagt senere
- ( ) barnevernet har åpnet undersøkelse, men ikke gitt tilbakemelding om det er iverksatt tiltak eller om saken er henlagt
- ( ) barnevernet har ikke åpnet undersøkelse. Saken henlagt
- ( ) barnevernet har ikke gitt noen tilbakemelding
- ( ) annet

vennligst noter

- ( ) vet ikke

De neste spørsmålene omhandler den åttende bekymringsmeldingen du sendte fra og med 2012.

Hvilket kjønn hadde barnet på den åttende bekymringsmeldingen du sendte?

- jente
- gutt

Hvilken alder hadde barnet på den åttende bekymringsmeldingen du sendte?

- 0 - 3 år
- 4 - 7 år
- 8 - 11 år
- 12 - 15 år
- 16 -

Hadde den ene eller begge foreldrene til barnet du sendte åttende bekymringsmelding på utenlandsk opprinnelse?

- ☐ ja
- ☐ nei
- ☐ vet ikke

Hva var årsaken til den åttende bekymringsmeldingen du?
Flere valg er mulig.

- ☐ mistanke om fysisk mishandling
- ☐ mistanke om seksuelt misbruk
- ☐ mistanke om psykisk mishandling
- ☐ mistanke om vanskjøtsel
- ☐ gjentagende ikke møtt til time
- ☐ grav karies
- ☐ gingivitt
- ☐ mangelfull hygiene
- ☐ sår og lesjoner i munnhulen
- ☐ traume
- ☐ andre orale funn

vennligst noter

- ☐ behandlingsvegring
- ☐ samspill med foresatte
- ☐ unormal oppførsel hos barnet
- ☐ annet

vennligst noter

Hvilken tilbakemelding har du fått fra barnevernet på den åttende bekymringsmeldingen du sendte?

- ○ barnevernet har åpnet undersøkelse, og iverksett tiltak
- ○ barnevernet har åpnet undersøkelse, men henlagt senere
- ○ barnevernet har åpnet undersøkelse, men ikke gitt tilbakemelding om det er iverksatt tiltak eller om saken er henlagt
- ○ barnevernet har ikke åpnet undersøkelse. Saken henlagt
- ○ barnevernet har ikke gitt noen tilbakemelding
- ○ annet

vennligst noter

- ○ vet ikke

De neste spørsmålene omhandler den niende bekymringsmeldingen du sendte fra og med 2012.

Hvilket kjønn hadde barnet på den niende bekymringsmeldingen du sendte?

- jente
- gutt

Hvilken alder hadde barnet på den niende bekymringsmeldingen du sendte?

- 0 - 3 år
- 4 - 7 år
- 8 - 11 år
- 12 - 15 år
- 16 -

Hadde den ene eller begge foreldrene til barnet du sendte niende bekymringsmelding på utenlandsk opprinnelse?

- ☐ ja
- ☐ nei
- ☐ vet ikke

Hva var årsaken til den niende bekymringsmeldingen du sendte?
Flere kategorier kan velges.

- ☐ mistanke om fysisk mishandling
- ☐ mistanke om seksuelt misbruk
- ☐ mistanke om psykisk mishandling
- ☐ mistanke om vanskjøtsel
- ☐ gjentagende ikke møtt til time
- ☐ grav karies
- ☐ gingivitt
- ☐ mangelfull hygiene
- ☐ sår og lesjoner i munnhulen
- ☐ traume
- ☐ andre orale funn

vennligst noter

- ☐ behandlingsvegring
- ☐ samspill med foresatte
- ☐ unormal oppførsel hos barnet
- ☐ annet

vennligst noter

Hvilken tilbakemelding har du fått fra barnevernet på den niende bekymringsmeldingen du sendte?

- ○ barnevernet har åpnet undersøkelse, og iverksett tiltak
- ○ barnevernet har åpnet undersøkelse, men henlagt senere
- ○ barnevernet har åpnet undersøkelse, men ikke gitt tilbakemelding om det er iverksatt tiltak eller om saken er henlagt
- ○ barnevernet har ikke åpnet undersøkelse. Saken henlagt
- ○ barnevernet har ikke gitt noen tilbakemelding
- ○ annet

vennligst noter

- ○ vet ikke

De neste spørsmålene omhandler den tiende bekymringsmeldingen du sendte fra og med 2012.

Hvilket kjønn hadde barnet på den tiende bekymringsmeldingen du sendte?

- jente
- gutt

Hvilken alder hadde barnet på den tiende bekymringsmeldingen du sendte?

- 0 - 3 år
- 4 - 7 år
- 8 - 11 år
- 12 - 15 år
- 16 -

Hadde den ene eller begge foreldrene til barnet du sendte tiende bekymringsmelding på utenlandsk opprinnelse?

- ja
- nei
- vet ikke

Hva var årsaken til den tiende bekymringsmeldingen du sendte?

Flere kategorier kan velges.

- ☐ mistanke om fysisk mishandling
- ☐ mistanke om seksuelt misbruk
- ☐ mistanke om psykisk mishandling
- ☐ mistanke om vanskjøtsel
- ☐ gjentagende ikke møtt til time
- ☐ grav karies
- ☐ gingivitt
- ☐ mangelfull hygiene
- ☐ sår og lesjoner i munnhulen
- ☐ traume
- ☐ andre orale funn

vennligst noter

- ☐ behandlingsvegring
- ☐ samspill med foresatte
- ☐ unormal oppførel hos barnet
- ☐ annet

vennligst noter

Hvilken tilbakemelding har du fått fra barnevernet på den tiende bekymringsmeldingen du sendte?

- ○ barnevernet har åpnet undersøkelse, og iverksett tiltak
- ○ barnevernet har åpnet undersøkelse, men henlagt senere
- ○ barnevernet har åpnet undersøkelse, men ikke gitt tilbakemelding om det er iverksatt tiltak eller om saken er henlagt
- ○ barnevernet har ikke åpnet undersøkelse. Saken henlagt
- ○ barnevernet har ikke gitt noen tilbakemelding
- ○ annet

vennligst noter

- ○ vet ikke

Har du noe du ønsker å kommentere når det gjelder bekymringsmeldinger?

Vennligst utdyp

I løpet av den tiden du har arbeidet som tannhelsepersonell, har du noen gang unnlatt å sende bekymringsmelding selv om du har hatt mistanke om barnemishandling eller omsorgssvikt?

- ja

Omtrent hvor mange ganger har du unnlatt å sende bekymringsmelding? Skriv inn siffer.

- nei

Hva var grunnene til at du unnlot å sende bekymringsmelding til barnevernet i de tilfellene du hadde mistanke om barnemishandling eller omsorgssvikt?

	Stemmer helt	Stemmer litt	Stemmer ikke	Vet ikke
var usikker på egne vurderinger				
hadde ingen å diskutere bekymringen med				
hindret av taushetsplikt				
hadde ikke nok kunnskap om barnemishandling og omsorgssvikt				
var usikker på hvordan en sender bekymringsmelding				
var usikker på hvor bekymringsmeldingen skulle sendes				
var usikker på hvordan en skulle dokumentere funnene/ mistanken				
var redd for hva som ville skje med barnet				
var redd for hva som ville skje med foreldrene				
var redd for hvilke konsekvenser det ville få for familien				
var redd for foreldrenes reaksjon				
var redd for å få trusler				
var redd for hvordan barnevernet ville håndtere bekymringsmeldingen				
ubehagelig å melde siden en ikke kan være annonym				
var redd for at barnet ville slutte å gå til tannklinikken				
var redd for tannklinikken sitt rykte i lokalsamfunnet				
manglet støtte fra klinikksjef (evt nærmeste leder)				
hadde ikke rutiner for å sende bekymringsmeldinger				
annet				

Hvilke andre grunner hadde du for å unnlate å melde?

Vennligst kommenter.

De neste spørsmål omhandler dine erfaringer med opplæring innen tematikken barnemishandling og omsorgssvikt.

Fikk du opplæring i tematikken barnemishandling, omsorgssvikt og meldeplikt til barnevernet under din utdanning?

- ja
- nei
- vet ikke

Fra og med 2012, hvilke år har du jobbet som tannhelsepersonell i den offentlige tannhelsetjenesten?

Kryss av for årene du har jobbet som tannhelsepersonell i den offentlige tannhelsetjenesten.

- ☐ 2012
- ☐ 2013
- ☐ 2014

Fra og med 2012 og frem til i dag, har du i forbindelse med ditt arbeid som tannhelsepersonell deltatt på opplæring/kurs/samarbeidsmøter relatert til tematikken barnemishandling, omsorgssvikt og barnevern?

Dersom ja, omtrent hvor mange dager?

- ja, 1 dag eller mindre
- ja, 2 - 4 dager
- ja, 5 dager eller mer
- nei
- vet ikke

Hvor dårlig eller god var kvaliteten på opplæringen du har fått fra og med 2012 og frem til i dag?

- veldig dårlig
- dårlig
- litt dårlig
- verken dårlig eller god
- litt god
- god
- veldig god

Har du behov for mer opplæring på rutiner for å sende bekymringsmeldinger til barnevernet?

- nei, ikke behov for mer opplæring
- usikker
- ja, har behov for litt mer opplæring
- ja, har behov for mer opplæring
- ja har behov for mye mer opplæring

Har du behov for mer opplæring innen temaet barnemishandling og omsorgssvikt?

- nei, ikke behov for mer opplæring
- usikker
- ja, har behov for litt mer opplæring
- ja, har behov for mer opplæring
- ja har behov for mye mer opplæring

Har du kjennskap til hvem som arbeider i det kommunale barnevernet i kommunen der tannklinikken er lokalisert ?

- ja
- nei
- vet ikke

Har du fått kjennskapen om hvem som jobber i barnevernet gjennom din jobb som tannhelsepersonell?

- ja
- nei
- vet ikke

Vennligst kryss av for de følgende spørsmål om rutiner og samarbeid med barnevernet.

	ja	nei	vet ikke
Har din tannklinikk skriftlig samarbeidsavtale med det lokale barnevernet?	○	○	○
Har du i forbindelse med ditt arbeid deltatt på møte med barnevernet?	○	○	○
Har din tannklinikk skriftlige rutiner for hvordan en skal sende bekymringsmeldinger?	○	○	○
Har din tannklinikk brevmal for sending av bekymringsmelding til barnevernet?	○	○	○
Har tannhelsetjenesten i ditt fylke etablert egen ressursperson/ressursteam knyttet opp til tematikken barnemishandling, omsorgssvikt og bekymringsmelding?	○	○	○

Hvordan er rutinene for sending av bekymringsmelding på din klinikk, hvem har ansvar for å sende bekymringsmeldingen?

- hver enkelt har ansvar for å sende sin bekymringsmelding
- klinikksjef sender alle bekymringsmeldingene
- sekretær sender alle bekymringsmeldingene
- annet

vennligst kommenter

Du har vært i kontakt med barnevernet i forbindelse med bekymringsmelding. Sett under ett, hvor dårlig eller god opplevde du...

	veldig dårlig	dårlig	verken dårlig eller god	god	veldig god
barnevernets veiledning underveis i meldeprosessen?					
barnevernets forståelse av tannklinikkens bekymring?					
barnevernets tilbakemelding til tannklinikken om utfallet av meldingene?					

Fra og med 2012 og frem til i dag, har du mottatt forespørsler fra barnevernet på pasienter som er under utredning hos barnevernet?

- ja

vennligst før opp hvor mange forespørsler du har mottatt fra barnevernet? Skriv inn siffer

- nei

De neste spørsmål omhandler din generelle oppfatning av barnevernet.

Hvor uviktig eller viktig samarbeidspart mener du at barnevernet er for tannhelsetjenesten?

Som samarbeidspart er barnevernet...

- veldig uviktig
- uviktig
- verken viktig eller uviktig
- litt viktig
- veldig viktig

Hvilken mistillit eller tillit har du til barnevernet?

Til barnevernet har jeg...

- fullstendig mistillit
- mistillit
- verken tilitt eller mistillit
- tilitt
- fullstendig tillit

Hvor lukket eller åpent opplever du som tannhelsepersonell barnevernet?

Jeg opplever barnevernet som...

- helt lukket
- lukket
- verken åpent eller lukket
- åpent
- helt åpent

Hvilken mangel på respekt eller respekt har du når det gjelder barnevernet?

Når det gjelder barnevernet har jeg...

- stor mangel på respekt
- mangel på respekt
- verken respekt eller mangel på respekt
- respekt
- stor respekt

Sett under ett hvor misfornøyd eller fornøyd er du med barnevernet?

Med barnevernet er jeg...

- veldig misformøyd
- misfornøyd
- verken fornøyd eller misfornøyd
- fornøyd
- veldig fornøyd

Dersom du har noen utfyllende kommentarer på samarbeidet med barnevernet vennligst kommenter.

Kommentar

Din tannklinikk har samarbeidsavtale med barnevernet. De neste spørsmål omhandler samarbeidsavtalen.

Har samarbeidsavtalen bidratt til å gjøre det vanskeligere eller enklere for deg å kontakte barnevernet?

Samarbeidsavtalen har gjort det å kontakte barnevernet...

- mye vanskeligere
- vanskeligere
- verken enklere eller vanskeligere
- enklere
- mye enklere

Har samarbeidsavtalen bidratt til å etablere mistillitt eller tillit til barnevernet?

For meg har samarbeidsavtalen har bidratt til å etablere...

- fullstendig mistillit
- mistillit
- verken tillit eller mistillit
- tillit
- fullstendig tillit

Opplever du at samarbeidsavtalen har redusert eller økt den gjensidige forståelsen for hvordan barnevernet og tannhelsetjenesten arbeider?

Samarbeidsavtalen har ført til...

- stor reduksjon i forståelsen
- reduksjon i forståelsen
- verken økt eller redusert forståelse
- økning i forståelsen
- stor økning i forståelsen

Tannklinikken kan få forespørsler fra barnevernet på barn som er under utredning. Opplever du at samarbeidsavtalen har bidratt til en reduksjon eller økning i antall forespørsler fra barnevernet?

Jeg opplever at samarbeidsavtalen har bidratt til...

- stor reduksjon i antall forespørsler
- reduksjon i antall forespørsler
- verken økt eller redusert antall forespørsler
- økning i antall forespørsler
- stor økning i antall forespørsler

Hvor misfornøyd eller fornøyd er du med samarbeidsavtalen?

Med samarbeidsavtalen er jeg...

- veldig misformøyd
- misfornøyd
- verken fornøyd eller misfornøyd
- fornøyd
- veldig fornøyd

Hvor dårlig eller godt kjenner du til innholdet i samarbeidsavtalen med barnevernet?

Jeg kjenner innholdet i samarbeidsavtalen...

- veldig dårlig
- dårlig
- verken godt eller dårlig
- godt
- veldig godt

De neste spørsmål omhandler tannklinikkens skriftlige rutiner for sending av bekymringsmelding

Din tannklinikk har skriftlige rutiner for når og hvordan en sender bekymringsmelding til barnevernet. Har rutinene gjort deg mer usikker eller tryggere på å sende bekymringsmelding?

Rutinene har gjort meg...

- mye mer usikker
- usikker
- verken sikker eller usikker
- sikrere
- mye sikrere

Din tannklinikk har skriftlige rutiner for å sende bekymringsmelding. Har rutinene gjort det vanskeligere eller enklere å sende en bekymringsmelding?

Jeg opplever at rutinene har gjort det...

- mye vanskeligere
- vanskeligere
- verken enkelere eller vanskeligere
- enklere
- mye enklere

Din tannklinikk har skriftlige rutiner for sending av bekymringsmelding. Har rutinene ført til økt eller redusert belastning for tannhelsepersonell?

Jeg opplever at rutinene har ført til...

- veldig økt belastning
- økt belastning
- verken økt eller reduser belastning
- redusert belastning
- veldig redusert belastning

Din tannklinikk har skriftlige rutiner for sending av bekymringsmelding. Har rutinene bidratt til å svekke eller øke din bevissthet i forhold til helsepersonell sin meldeplikt til barnevernet?

Rutinene har bidratt til å...

- svekke min bevissthet mye
- svekke min bevissthet
- verken økt eller svekket min bevissthet
- økt min bevissthet
- økt min bevissthet mye

Hvor misfornøyd eller fornøyd er du med de skriftlige rutiner for sending av bekymringsmelding?

Med rutinene er jeg...

- veldig misformøyd
- misfornøyd
- verken fornøyd eller misfornøyd
- fornøyd
- veldig fornøyd

Hvor dårlig eller godt kjenner du rutinene for sending av bekymringsmelding?

Jeg kjenner rutinene...

- veldig dårlig
- dårlig
- verken godt eller dårlig
- godt
- veldig godt

Tannhelsetjenesten i ditt fylke har etablert egen ressursperson/ressursteam som er knyttet opp til tematikken barnemishandling, omsorgssvikt og barnevernet.

Opplever du det å kunne diskutere faglige funn og opplevelser med en ressursperson/ressursteam i egen organisasjon som unyttig eller nyttig?

Jeg opplever det å ha egen ressursperson/ressursteam som ...

- veldig unyttig
- unyttig
- verken nyttig eller unyttig
- nyttig
- veldig nyttig

De neste spørsmål omhandler helsepersonell sin taushetsplikt og meldeplikt.

Hvor usikker eller sikker er du ...

	veldig usikker	usikker	verken usikker eller sikker	sikker	veldig sikker
når det gjelder taushetsplikten og hvilke opplysninger du kan oppgi til barnevernet dersom du sender en bekymringsmelding?	◯	◯	◯	◯	◯
når det gjelder taushetsplikten og hvilke opplysninger du kan utgi dersom barnevernet henvender seg til deg angående en av dine pasienter?	◯	◯	◯	◯	◯
på taushetsplikten i mellom ulike tjenester (eks. tannklinikk, helsestasjon, barnehage, skole, barnevern)?	◯	◯	◯	◯	◯
i forhold til i hvilke tilfeller foresatte skal informeres om at du sender en bekymringsmelding?	◯	◯	◯	◯	◯

Hvor usikker eller sikker føler du deg...

	veldig usikker	usikker	verken usikker eller sikker	sikker	veldig sikker
på når en skal sende en bekymringsmelding?	◯	◯	◯	◯	◯
på hvordan en skal sende en bekymringsmelding?	◯	◯	◯	◯	◯
på innholdet i helsepersonell loven §33	◯	◯	◯	◯	◯

De neste spørsmål omhandler din bakgrunn.

I hvilket fylke er du ansatt?

- ◯ Aust Agder
- ◯ Buskerud
- ◯ Finnmark
- ◯ Hedmark
- ◯ Hordaland
- ◯ Møre og Romsdal
- ◯ Nordland
- ◯ Nord-Trøndelag
- ◯ Oppland
- ◯ Oslo
- ◯ Rogaland
- ◯ Sogn og Fjordane
- ◯ Sør-Trøndelag
- ◯ Telemark
- ◯ Troms
- ◯ Vest Agder
- ◯ Vestfold
- ◯ Østfold

Hva er din nåværende stilling i tannhelsetjenesten?

- tannpleier
- tannpleier med ledelsesoppgaver (klinikkleder, distriktsleder, direktør)
- tannlege
- spesialisttannlege
- tannlege med ledelsesoppgaver (klinikkleder, distriktsleder, overtannlege, direktør, fylkestannlege)
- annet

vennligst spesifiser

Hvor mange år har du vært tilsatt i den offentlige tannhelsetjenesten?

Vennligst oppgi hele år. Dersom det er mindre enn ett år, skriv inn 0

År tilsatt i tannhelsetjenesten.

Skriv inn siffer

Hvilket kjønn er du?

- kvinne
- mann

Hvilken alderskategori tilhører du?

- 20 - 29 år
- 30 - 39 år
- 40 - 49 år
- 50 - 59 år
- 60 - 69 år
- 70 -

Totalt hvor mange tilsette (tannhelsesekretærer, tannpleiere og tannleger) er det på din tannklinikk?

Dersom du jobber på flere steder, oppgi antall på den klinikken du jobber mest.

- 1-3
- 4-6
- 7-9
- 10-14
- 15-19
- 20-24
- 25-29
- 30+

Hvor mange innbyggere er det i kommunen tannklinikken er lokalisert?

Dersom du jobber ved flere tannklinikker vennligst oppgi for den klinikken du jobber mest.

- ◯ 0 - 5000
- ◯ 5001 - 10.000
- ◯ 10.001 - 15.000
- ◯ 15.001 - 20.000
- ◯ 20.001 - 40.000
- ◯ 40.001 - 80.000
- ◯ 80.001+

Omtrent hvor mange pasienter under 18 år har du undersøkt eller behandlet siste 12 måneder?

- ◯ 0 - 250
- ◯ 251 - 500
- ◯ 501 - 750
- ◯ 751 - 1000
- ◯ 1001 - 1250
- ◯ 1251 - 1500
- ◯ 1501 +

Har du andre kommentarer relatert til tema "tannhelse og barnevern" kan du gjerne utdype dette her:

Kommentar

Hjertelig takk for din deltakelse.

Spørreundersøkelsen er nå fullført.

Takk!

UNIVERSITETET I BERGEN
HEMIL-senteret
Senter for forskning om helsefremmende arbeid, miljø og livsstil

Tannhelsetjenestens kompetansesenter Vest / Hordaland

Kontakt: Ingfrid Vaksdal Brattabø E-mail: ingfrid.brattabo@hfk.no
Adresse: Tannhelsetjenestens kompetansesenter vest, Hordaland, Pb. 2354 Møllendal, Bergen 5867, Norway
